# Promoting the application of *Pinus thunbergii* Parl. to enhance the growth and survival rates of post-germination somatic plantlets

**DOI:** 10.1186/s12870-023-04175-1

**Published:** 2023-04-12

**Authors:** Tingyu Sun, Yanli Wang, Xiaoqin Wu, Jianren Ye, Fang Cheng

**Affiliations:** 1 Innovation Center of Sustainable Forestry in Southern China, Collaborative College of Forestry, Nanjing Forestry University, Nanjing, 210037 Jiangsu China; 2 Jiangsu Key Laboratory for Prevention and Management of Invasive Species, Nanjing Forestry University, Nanjing, 210037 Jiangsu China; 3 Anhui Agricultural University, Anhui, China; 4 Management Bureau of Guangdong Xiangtoushan National Nature Reserve, Huizhou, China

**Keywords:** Afforestation, Acclimation, Growth factors, *Pinus thunbergii*, Somatic plants

## Abstract

**Objective:**

There is a growing need for nematode resistant Pinaceae species plantlets to cope with the global scale degradation of coniferous forests, due to the prevalence of pine wilt disease. One of the bottlenecks that limits the commercialization of Pinaceae species plantlets is regeneration following their transfer from controlled sterile environments to the field while maintaining high survival rates.

**Methods:**

The growth factors of somatic plantlets (SPs), such as sucrose, media, culture substrate, brassinolide and spectrum were investigated to promote the application of somatic nematode-resistant *P. thunbergii* plants in afforestation.

**Results:**

The 1/2 WPM liquid medium, culture substrate (perlite and vermiculite =1:1), and carbohydrate (20 g/L sucrose) were effective in stimulating the growth of rooted SPs. While for unrooted SPs, 1 ug/L of brassinolide enhanced plantlet growth and rooting. And blue light (B) significantly promoted the longitudinal growth of shoots, while red light (R) was beneficial for root growth during the laboratory domestication stage. High quality SPs were obtained at a R/B ratio of 8:2. Following this acclimatization protocol, the *P. thunbergii* SPs could be directly transplanted to the field with a higher survival rate (85.20 %) in a forcing house.

**Conclusion:**

this acclimatization protocol extremely improved the survival rate of *P. thunbergii* SPs. Moreover, this work will contribute to enhancing the possibilities for somatic plant afforestation with *Pinus* species.

**Supplementary Information:**

The online version contains supplementary material available at 10.1186/s12870-023-04175-1.

## Introduction

*Pinus thunbergii* is an evergreen tree species that is resistant to sea mist and wind; thus, it is extensively employed in coastal urban hill greening, and as a coastal windbreak in China, Japan, and Southern Korea [1–3]. However, it is susceptible to pine wilt disease caused by the pine wood nematode (*Bursaphelenchus xylophilus*), which has propagated worldwide. The epidemic of pine wilt disease has led to a massive decline of *P. thunbergii* trees and threatened entire *Pinus* ecosystems [4]. Thus, the deployment of PWN resistant genotypes is an important strategy for controlling the disease [5]. A long-term selective breeding project has been undertaken to develop PWN resistant coniferous trees (*P. thunbergii* and *P. densiflora*) in Japan [6–8]. Somatic embryogenesis has proven to be one of the most promising techniques for the mass propagation of conifers. Many regenerated plantlets had been obtained from *Pinus* species via somatic embryogenesis, including *P. pinea* [9], *P. thunbergii* [10, 11], *P. radiata* [12], and *P. elliottii* [13]. However, reports on the application of somatic plants for afforestation are rare, where low survival rates are the primary challenge [14].

Differences between the microenvironments of seedlings cultured in the lab with those in field leave regenerated plantlets with delicate and weakened root systems, which results in low survival rates of ginger [15]. In Medicago arborea L. study, an effective acclimatization project might enable the generation of high-quality seedlings to improve their survival rates [16]. As a plant growth facilitator, sugar is essential as it plays a key role in molecular signaling processes during plant development [17]. During micropropagation, sucrose-cleaving enzymes rapidly promotes leaf growth. Moreover, the transport of sugars influences the development of aboveground organs and roots in Arabidopsis and *Saccharum* species [18–20]. Therefore, the application of sugars in vitro is a key factor toward enhancing the growth and development of plantlets [21, 22]. Culture mediums and substrates provide essential nutrients, and possess good water retention, air permeability, and acid-base buffering capacities for plant regeneration. Their appropriate selection is one of the essential factors in determining the survival rates of transplants. In the study of *P. densiflora*, the survival rate of plantlets was up to 60% in a mixture of culture substrate containing vermiculite-perlite-sand (1:1:1) [23].

Brassinosteroids (BRs) have been extensively applied in plant growth studies, which are considered to be effective and ecofriendly phytohormones that regulate the differentiation of root epidermal cells, while facilitating the formation of lateral roots [24, 25]. Spectral treatments that affected plant physiology have been demonstrated in horticultural species [26, 27]. Additional knowledge on the responses of conifer plantlets to different spectra is required to develop commercial applications to produce high-quality plantlets for economically viable and efficient forest regeneration [28]. Recently, the beneficial effects of spectral treatments on plant growth have been widely observed on horticultural species [29, 30]. These factors justify the enormous efforts that have been undertaken to improve nursery protocols for the generation of highly quality plantlets. However, these studies, particularly the application of BRs and spectral treatments for the regulation of plant growth, have been less frequent for *Pinus* species [31].

Previously, our group obtained the regenerated plantlets of nematode-resistant *P. thunbergii* through somatic embryogenesis [32, 33]. To further promote the application of somatic nematode-resistant *P. thunbergii* plants in afforestation, we studied the effects of sucrose, medium types, BRs, and spectral treatments on the growth of SPs. Furthermore, their survival rates were continuously monitored. The aim of this study was to develop an effective acclimatization cultivation project to increase the survival rate of nematode-resistant *P. thunbergii*. This study contributes to improving the possibility of somatic plant afforestation with *Pinus* species.

## Materials and methods

### Plant material

The SPs were obtained from 1539 to 1 and 1637-2 cell lines through somatic embryogenesis. The 1539-1 and 1637-2 cell lines were initiated from nematode-resistant families 39 and 37 of *P. thunbergii*, respectively. The nematode-resistant families were introduced from Japan (average survival rates of nematode-resistant *P. thunbergii* were 51%, which was 35% higher than for unselected populations) [34, 35], where the seedings from families 37 and 39 showed a high resistance to *Bursaphelenchus xylophilus* in a previous report [36]. Here, the resistance of maternal trees will continue to be monitored in an experimental field of Nanjing Forestry University (Jiangsu, China).

**Fig. 1:**
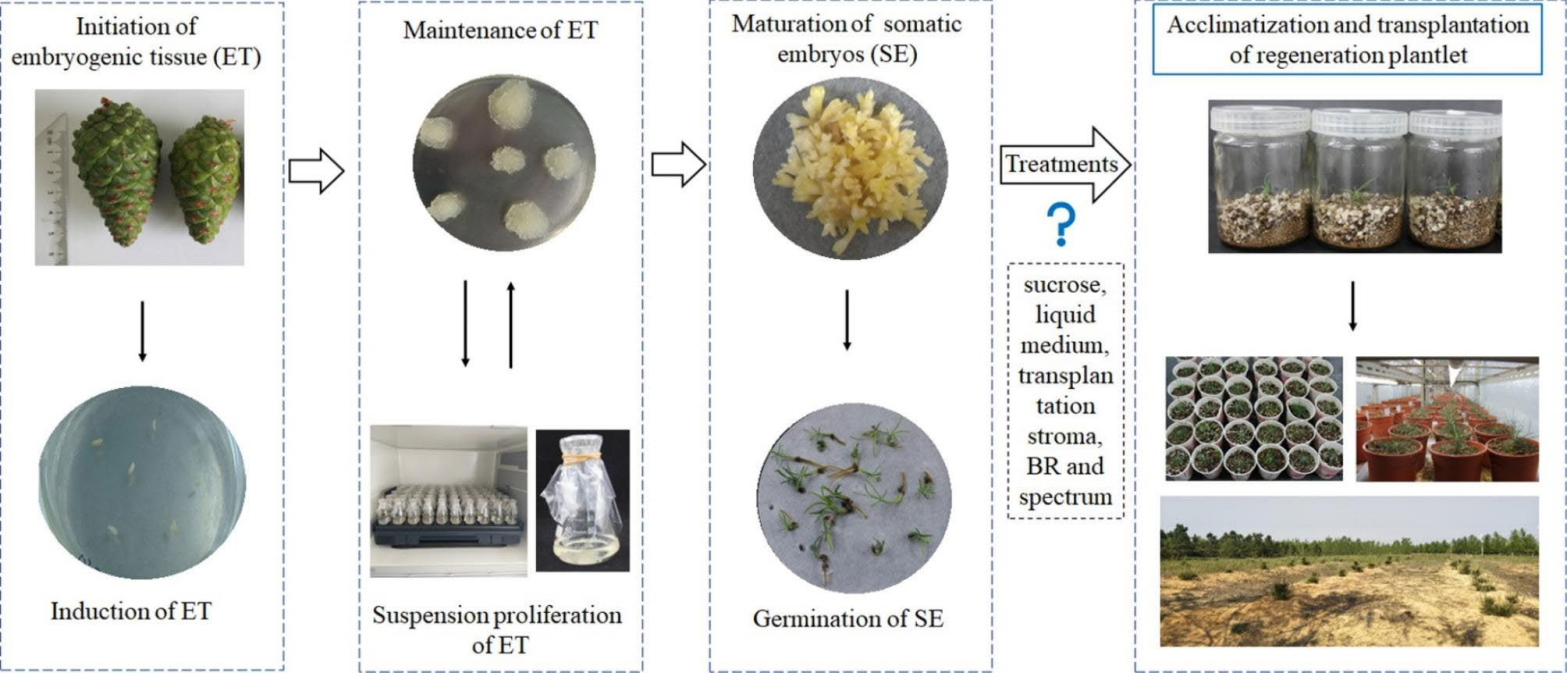
Plant regeneration via somatic embryogenesis in nematode-resistant *Pinus thunbergii*

Protocols for the somatic embryogenesis of nematode-resistant *P. thunbergii* were conducted according to our previous report [32, 33]. Briefly, embryogenic cells were initiated from the whole megagametophyte (at about the cleavage poly-embryo stage) from immature cones collected from the Nanjing Forestry University gene pool of nematode-resistant pines (*P. thunbergii*), in Jiangsu, China. The initiated embryogenic cells were cultured on a maintenance medium for proliferation. Subsequently, the embryogenic cells (fresh weight was 1 g in petri dish) were transferred to 100 mL flasks (including a 30 mL liquid medium), and the suspensions were placed on gyratory shaker at 90 rpm, and cultured for 1 week. The 2 mL suspensions were transferred to the maturation medium for 10 weeks at 25 ± 1 ℃ in the dark. After 10 weeks (without intervals), the cotyledon embryos were taken from the maturation medium and transferred to the germination medium. The cotyledon embryos were precultured on a germination medium for 1 week and then placed under 16/8 h light conditions for four weeks at 25 ± 1℃. The germinated somatic embryos were selected as experimental samples for acclimation tests for this paper (Fig. 1).

The maturation medium consisted of a Lobbly pine medium (LP) [37]. The basic medium contained 3% (w/v) maltose; 500 mg/L casein acid hydrolysate; 10 mg/L abscisic acid; 1 g/L myo-inositol; 2 g/L activated charcoal; 130 g/L polyethylene glycol 8000 (PEG 8000); 1500 mg/L L-glutamine (filtering sterilization); and 3.5 g/L phytagel.

The germination medium comprised a half-strength (large element) LP basic medium supplemented with 20 g/L sucrose, 2 g/L activated charcoal, and 8 g/L agar. Prior to sterilization, the pH of all media was adjusted to 5.8 and autoclaved (121℃, 20 min).

### Determination of root and shoot lengths after sucrose, liquid medium, and culture substrate treatments

The SPs of the 1539-1 rooted on the germination medium were transferred to the growth medium. The SPs were cultured on the growth medium for one-month under cool white fluorescent light (16/8 h) at 25 ± 1℃. For all treatments, the group was treated with 15 replicates and repeated three times. The culture substrate was bottled for sterilization and cooled before adding the sterilized liquid medium. Following growth enhancement, the somatic plants were transplanted into the field acclimatize in a culture room (forcing house).

#### Sucrose treatment

The growth medium was half-strength (large element) Woody Plant Medium [38] (1/2 WPM) liquid medium (30 mL), which was supplemented with culture substrate vermiculite: perlite = 1:1 (~ 1/5 volume of flask) and sucrose (10, 20, 30, and 40 g/L). Each SP was transferred into a dedicated flask (Ø6 cm x 9 cm high). The SP were cultured on the growth medium for one-month under standard conditions, after which they were removed from the flasks and the roots were rinsed. Next, the root and shoot length (total lengths of the aboveground parts) of the regenerated plants were measured with a scale.

#### Liquid medium treatment

Four basal media were selected for this experiment. These media included Gupta and Durzan medium (DCR) medium [39]; Lobbly pine medium (LP) medium [37]; Gresshoff and Doy (GD) medium [40]; and Woody Plant Medium (WPM) medium [38]. Four basic media components were listed (Table S1). The growth medium was supplemented with sucrose (20 g/L), culture substrate vermiculite: perlite = 1:1 (~ 1/5 volume of flask) and the liquid medium (DCR, LP, GD and 1/2 WPM) (30 mL). The other conditions were the same as the sucrose treatments.

#### Culture substrate treatment

The growth medium contained 30 mL liquid medium (1/2 WPM), which was supplemented with 20 g/L sucrose, and a 1/5 flask volume of culture substrate (perlite, vermiculite, perlite, and vermiculite = 1:1, respectively). The other conditions were identical to the sucrose treatments.

### Determination of root and shoot length following brassinolide and spectrum treatments

#### Brassinolide treatments

The SPs of the 1637-2 with shoots but no roots on the germination medium were transferred to the rooting medium. The rooting medium [41, 42] was supplemented with 1/4 WPM (large element reduction); brassinolide (0, 1 and 10 µg/L); 20 g/L sucrose; 0.5 g/L activated carbon; and 6 g/L agar. The SPs were cultured on the rooting medium for one-month under cool white fluorescent light (16/8 h) at 25 ± 1℃, and then removed. Each group was treated with 15 replicates and repeated three times. The root and shoot lengths (total lengths of the aboveground parts) were measured with a scale after rinsing three times.

#### Red and blue LED light treatments

Experiments that investigated the impacts of different light wavelengths on SPs growth were conducted with the 1637-2 cell lines. The SPs of the 1637-2 with shoots and roots on the germination medium were transferred to the growth medium (selected above). The growth medium was supplemented with 1/2 WPM liquid medium (30 mL), 20 g/L s sucrose, and 1/5 flask volume of culture substrate (perlite and vermiculite = 1:1, respectively). Each SP was transferred into a dedicated flask (Ø6 cm x 9 cm high). The plantlets were cultured for one-month at 25 ± 1℃. Each group was treated with 15 replicates and repeated three times. LEDs (Philips, Amsterdam, Netherlands, 100 µmol/m^2^/s) with different red and blue ratios were employed as the light sources (B indicates blue light (400–500 nm); 5R5B indicates red light: blue light = 5:5; 7R3B indicates red light: blue light = 7:3; 8R2B indicates red light: blue light = 8:2; R indicates red light (600–700 nm)). A cool white fluorescent lamp (Philips, Amsterdam, Netherlands, 100 µmol/m^2^/s) served as the CK. The root and shoot lengths (total lengths of the aboveground parts) were measured with a scale after rinsing. The rootstock ratio indicated the root length to shoot length (R/S) ratio. The root tips, volume, surface area and average diameter were measured using a root scanner (EPSON, V850PRO, China).

### Determination of survival rate

Following growth enhancement, the somatic plants were transplanted into a culture room for acclimatization. The transplanting substrate was mixture of pine forest soil, perlite, and vermiculite at a ratio of 2:1:1. Each somatic plant was transferred into a pot (Ø8 cm x 15 cm high). The somatic plants were cultured in culture room under cool white fluorescent light (16/8 h) at 25 ± 1℃, and watered once a week. The humidity of the culture room was maintained at above 85%. The survival rate of the plantlets was calculated after six months.

### Statistical analysis

The transplant survival rate was evaluated using an analysis of variance (ANOVA and Principal component analysis (PCA). The means were compared by Duncan’s honestly significant difference test at *p* < 0.05. All analyses were done using SPS version 19 (IBM, Armonk, NY, USA) and R (R Core Team 2019). Spearman’s correlation was utilized to analyze the relationships between the survival rates and root growth indicators. In addition, a binary logistic regression was used to analyse the main factors affecting somatic plant survival rate in light treatment.

## Results

### Enhancement of SPs between treatments

The sucrose concentration (shoot, *p* < 0.001; root, *p* = 0.002), liquid medium (shoot, *p* < 0.001; root, *p* < 0.001), and culture substrate (shoot, *p* = 0.03; root, *p* = 0.09) significantly impacted the SPs growth. Under the sucrose treatment, the SPs were significantly enhanced in 20 and 30 g/L sucrose. The needles of the SPs were sparse and yellow-green under a low concentration of sucrose (10 g/L), exhibiting an obvious nutrient deficiency. The SPs height (Shoot length, S = 2.05 ± 0.45 cm; Root length, R = 3.23 ± 0.48 cm) at a sucrose concentration of 20 g/L was significantly higher than the 10 g/L treatment (S = 1 ± 0.08 cm, R = 2.04 ± 0.34 cm). However, when the sucrose concentration was increased to 40 g/L, the growth of the SPs was inhibited (S = 0.58 ± 0.07 cm, R = 0.70 ± 0.18 cm). Under the culture medium treatment, the SPs obtained higher growth parameters in 1/2 WPM (S = 1.44 ± 0.14 cm, R = 4.47 ± 0.84 cm). In addition, for the *P. thunbergii* plantlets the 1/2 WPM and GD medium were superior to the DCR and LP medium. The growth of SPs fared the worst in the DCR medium (S = 0.54 ± 0.03 cm, R = 0.75 ± 0.19 cm), verifying that it was obviously not suitable for the growth of *P. thunbergii* SPs.

**Fig. 2:**
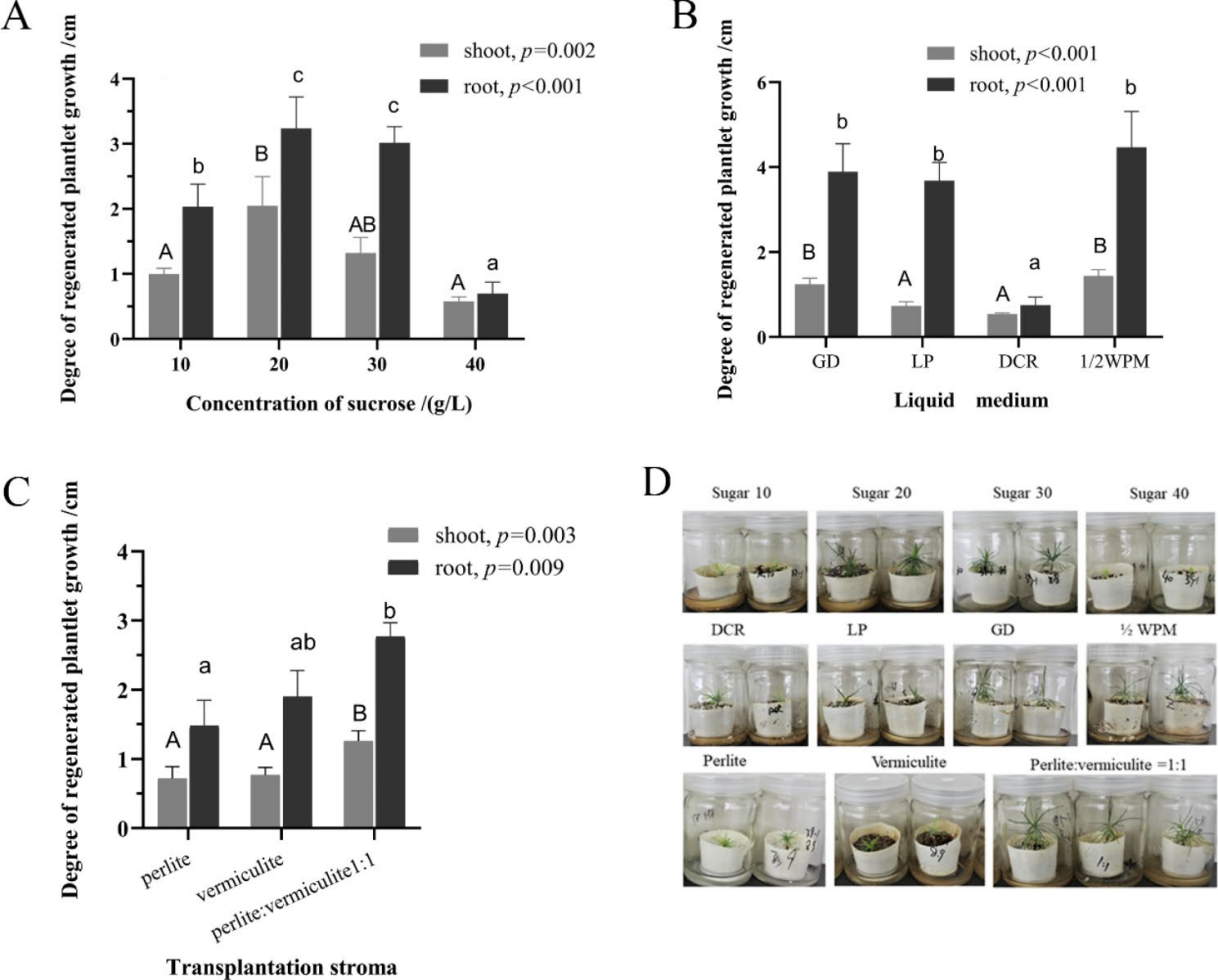
Effects of (**A**) sugar content, (**B**) liquid medium, and (**C**) culture substrate on growth of nematode-resistant *Pinus thunbergii* plantlets. **D**: Performance of plantlet growth in treatments. Data represent mean ± standard error (SE). Different letters indicate significant difference (p < 0.05 by Duncan’s test)

For the culture substrate treatments, the enhancement of the SPs in perlite and vermiculite = 1:1 treatment (S = 1.26 ± 0.15 cm, R = 2.77 ± 0.20 cm) was improved over that of the perlite only (S = 0.72 ± 0.17 cm, R = 1.48 ± 0.37 cm), and vermiculite (S = 0.77 ± 0.11 cm, R = 1.90 ± 0.38 cm). The lengths of the SPs shoots and roots cultured in a mixed substrate of perlite and vermiculite (v/v = 1:1) were significantly greater than when they were cultured in perlite and vermiculite alone. In general, the 20 g/L sucrose, 1/2 WPM liquid medium, and culture substrate (perlite: vermiculite = 1:1) were observed to significantly enhance the growth of nematode-resistant *P. thunbergii* SPs (Fig. 2).

### Effects of BR on the growth of SPs

**Fig. 3:**
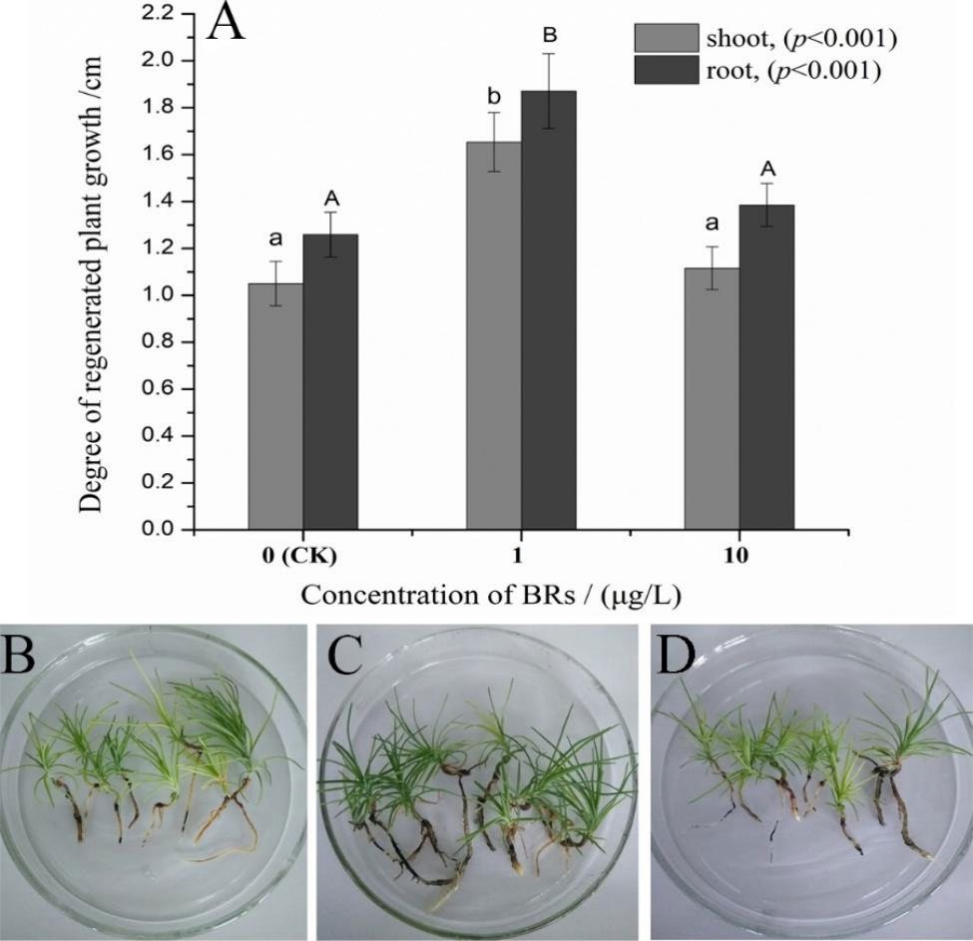
Effects of BR on growth of nematode-resistant *Pinus thunbergii* plantlets. **A**: Statistical analysis of root and shoot length of somatic plantlets. Data represent mean ± standard error (SE). **B**, **C**, and **D** indicate the performance of plantlet growth under 0, 1, and 10 µg/L BR treatments. Significant differences between variances were calculated by two-way ANOVA. Different letters indicate significant differences between roots and shoots (*p* < 0.05, by Duncan’s test)

The BR content significantly (*p* < 0.001) affected the growth of roots and shoots of the SPs without rooting. Statistical results revealed that the lengths of the roots and shoots of SPs under the 1 µg/L (S = 1.65 ± 0.13 cm, R = 1.87 ± 0.16 cm) BR treatment were significantly greater (*p* < 0.05) than those of the 10 µg/L BR (S = 1.11 ± 0.09 cm, R = 1.38 ± 0.09 cm), and the control group (S = 1.05 ± 0.10 cm, R = 1.26 ± 0.09 cm) (Fig. 3A). In the control group, the needles were light green and the root system consisted of only a single primary root without lateral roots. When the rooting medium was supplemented with 1 µg/L BR, the needles were dark green and a well-developed root system emerged (more lateral roots, greater root volume, and more root tips). However, when the BR concentration was increased to 10 µg/L, the SPs showed a similar differentiation to the control group (Fig. 3B, C, D). These results implied that the BR promoted growth of *P. thunbergii* SPs might have been related to root system differentiation and photosynthetic regulation.

### Effects of red and blue LED light sources on the growth of SPs

**Fig. 4:**
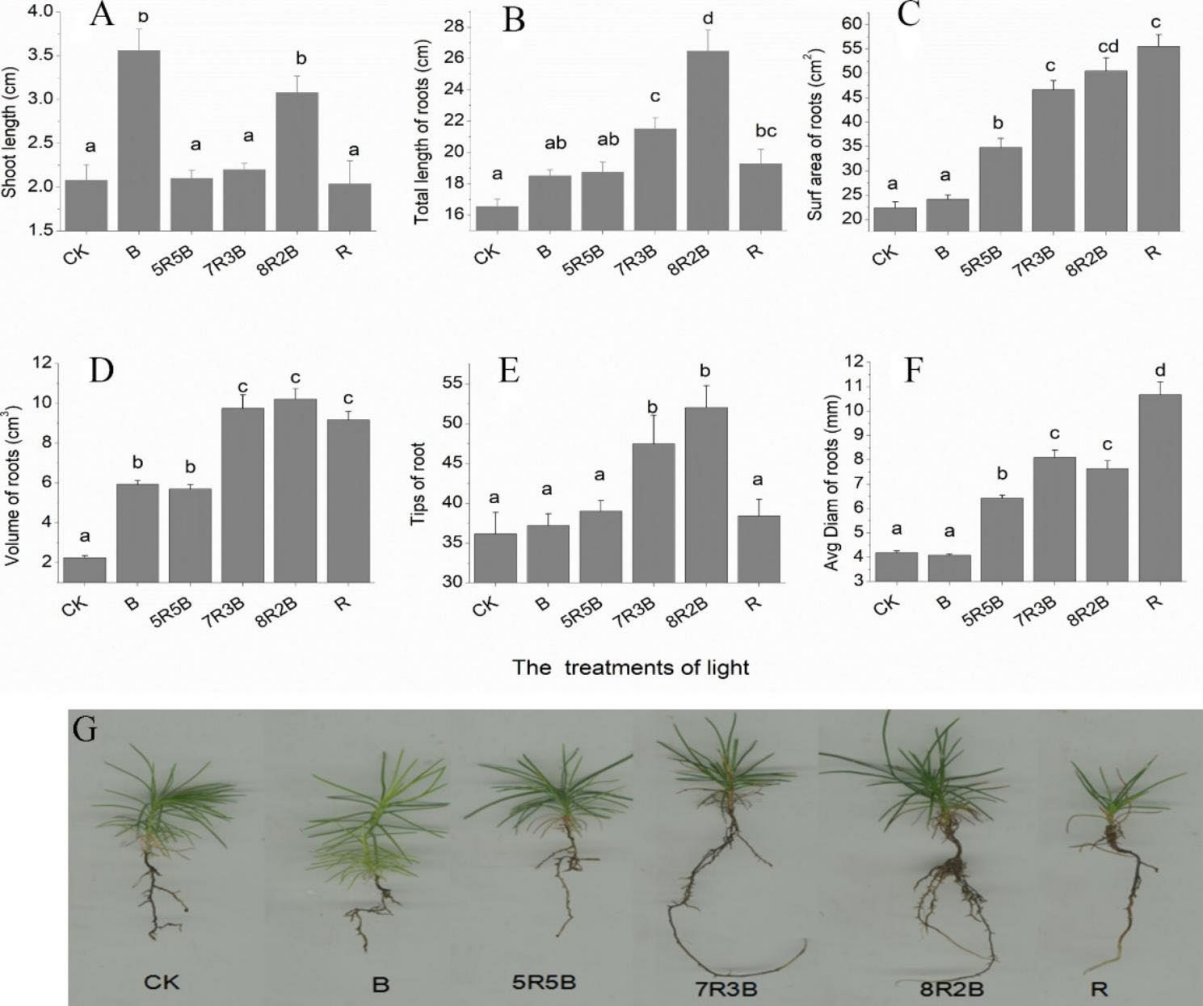
Effects of spectrum treatments on growth of (**A**) shoot length, (**B**) total length of roots, (**C**) surface area of roots, (**D**) volume of roots, (**E**) root tips, (**F**) average diameter of roots in nematode-resistant *Pinus thunbergii* plantlets. Data represent mean ± standard error (SE). Different lowercase letters indicate significant differences between spectrum treatments (*p* < 0.05, by Duncan’s test). **G**: Performance of plantlet growth under different light spectrum treatments. CK indicates cool white fluorescent; B indicates blue light; 5R5B indicates red light: blue light = 5:5; 7R3B indicates red light: blue light = 7:3; 8R2B indicates red light: blue light = 8:2; R indicates red light

The spectrum treatment had significant (*p* < 0.001) effects on the shoot length and root differentiation of the SPs. The greatest shoot length (3.56 cm) was obtained under the blue light treatment (Fig. 4A). In terms of root differentiation, the total lengths of roots, their volume, and the number of root tips all reached their peak under the 8R2B treatment (Fig. 4B, D, E). However, the root surface area and average diameter achieved their peaks under the R treatment (Fig. 4 C, F). The best performance of SPs was obtained under the 8R2B treatment (Fig. 4G). In general, blue light promoted the longitudinal growth of the shoots, whereas red light was beneficial for root development. Further, 8R2B was the optimal combination for the enhancement of the SPs.

### Survival rates of SPs

**Fig. 5:**
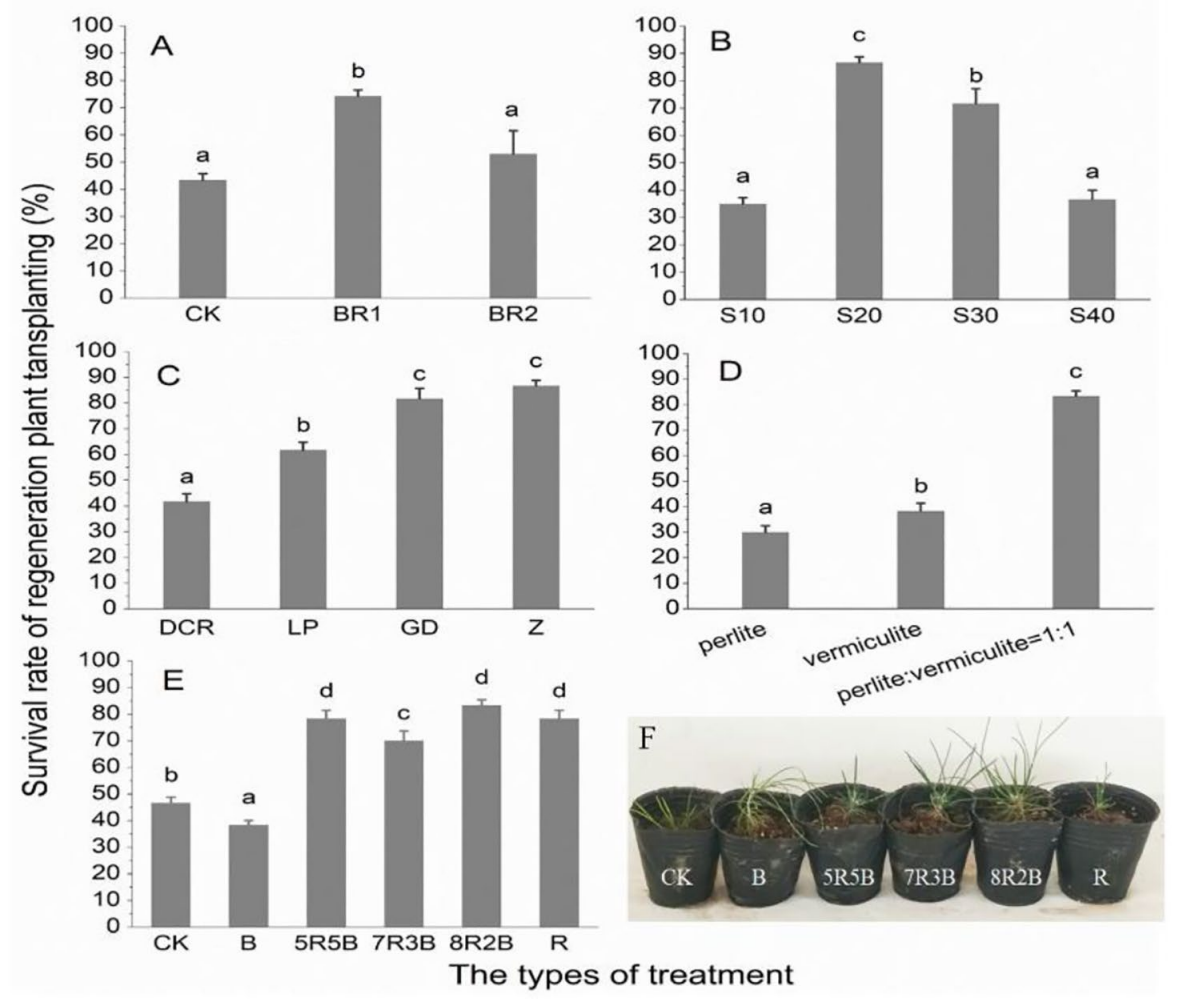
Survival rates of (**A**) BR, (**B**) sugar, (**C**) liquid medium, (**D**) culture substrate, (**E**) spectrum treatments of nematode-resistant *Pinus thunbergii* plantlets. (**F**) Performance of transplantation plantlets after spectrum treatments for one month. Data represent mean ± standard error (SE). Different lowercase letters indicate significant differences between spectrum treatments (*p* < 0.05, by Duncan’s test)

**Fig. 6:**
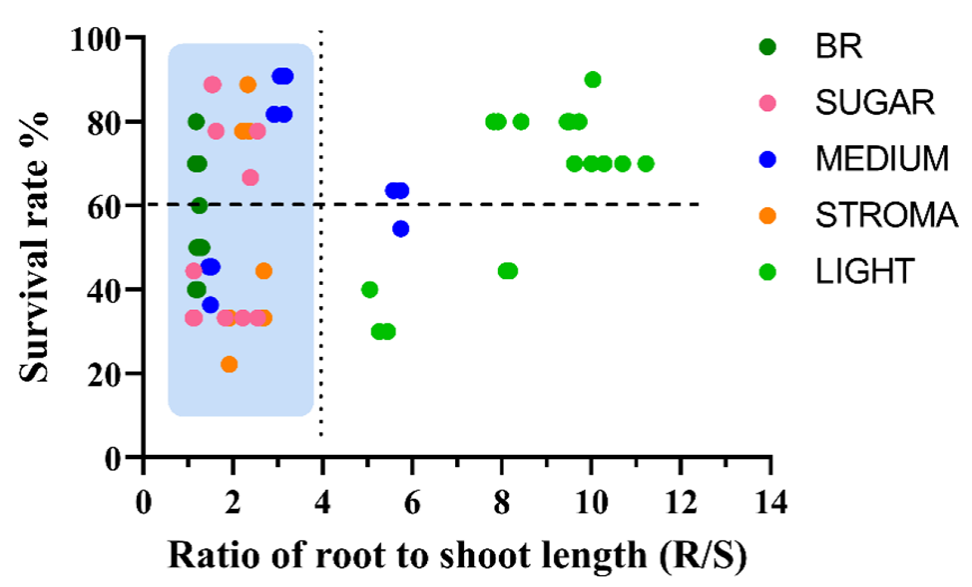
Effects of root to shoot length ratios on survival rate. BR: BR treatments, SUGAR; sugar content treatment, MEDIUM: liquid medium treatment, SUBSTRATE: culture substrate treatment, LIGHT: spectrum treatment. Light blue shading indicates root length < 5 cm. Dots represent the mean of each treatment

**Fig. 7:**
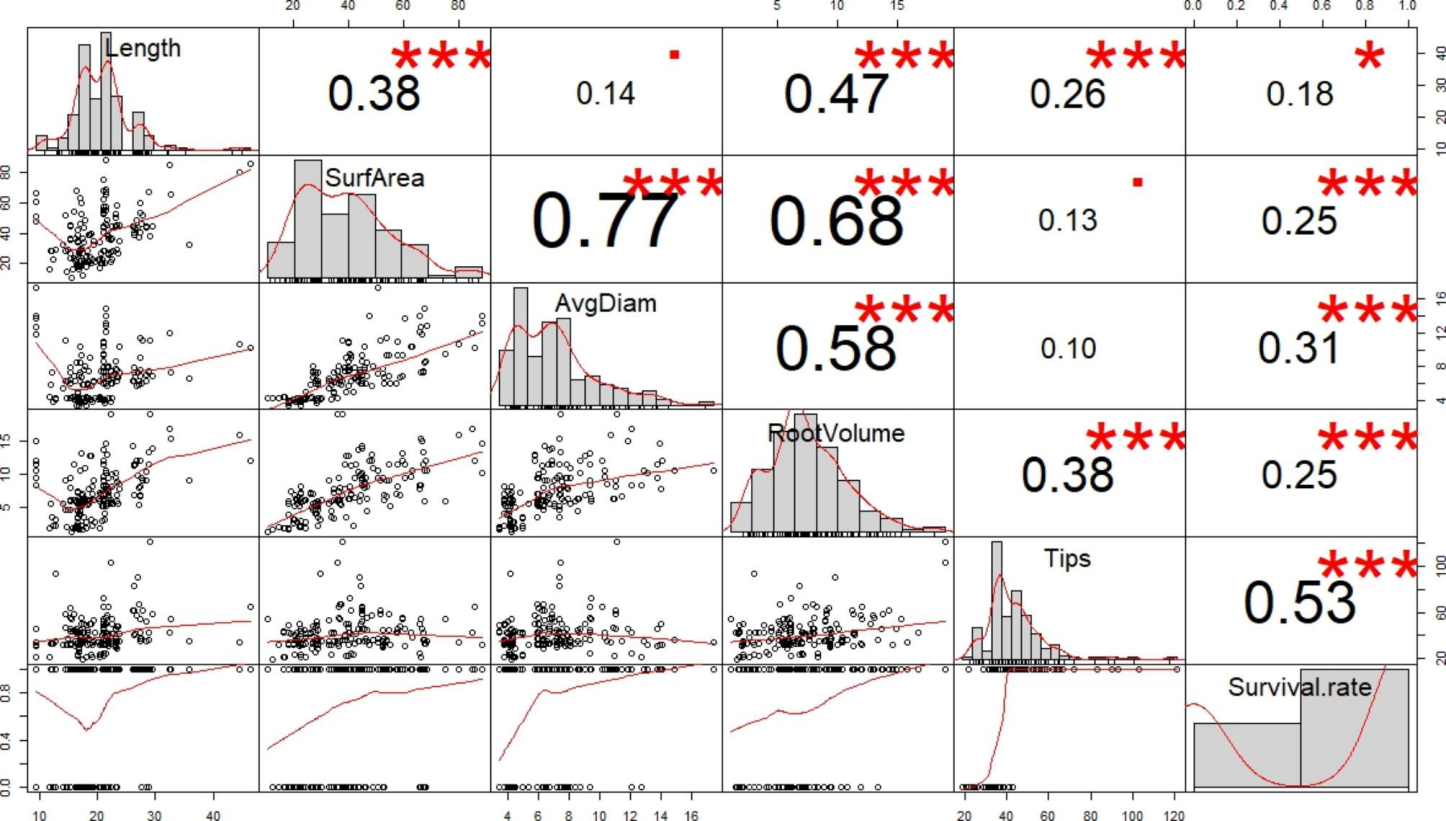
Correlation analysis of various factors affecting the survival rate. * and *** indicate significant differences at *p* < 0.05 and *p* < 0.001, respectively

The treatments that effectively promoted the growth of SPs also contributed to their survival rates. For example, the SPs treated with 20 g/L sucrose showed the highest survival rate (85.20%). Similarly, following the liquid medium and culture substrate treatments, the highest survival rate was obtained with the 1/2 WPM and mixture of vermiculite and perlite (1:1 (v/v)). Furthermore, the survival rate of SPs was also significantly (*p* < 0.05) promoted in the rooting medium with 1 µg/L BR (Fig. 5). Among the spectrum treatments, the highest survival rate occurred under the 8R2B, while the lowest survival rate occurred under the B treatment. As for the rootstock ratio (R/S), the results revealed that when the R/S was < 4 cm, there was no correlation with the survival rate. When the R/S was > 4 cm, it was positively related to the survival rate (Fig. 6). Among all treatments, the higher survival rates of the SPs were obtained under spectrum combinations. Based on the spectrum treatments, binary logistic regression showed that root length (*p* < 0.05), root tips (*p* < 0.001) and diameter (*p* < 0.001) significantly affected the survival rate of somatic plant, while the root volume (*p* = 0.072) and surface area (*p* = 0.528) were not significant (Table S3). Principal component analysis also showed that the factor that had the greatest influence on the survival rate was the tips (Fig. S1). Moreover, correlation analysis revealed that the correlation coefficient of the root tips on survival was the greatest (*p* < 0.001; r = 0.53), while the highest correlation with the root tips was the root volume (*p* < 0.001; r = 0.38). The root length had the lowest correlation coefficient (*p* < 0.05; r = 0.18) for the survival rate compared to the root tips, surface area, volume, and diameter (Fig. 7). This indicated that root length was not the key to the high survival rate of somatic plants compared to the root tip.

Binary logistic regression analysis further validated our hypothesis that medium (*p* = 0.0013), sugar (*p* < 0.001), substrate (*p* < 0.001), LED treatments (*p* < 0.001), and genotype (*p* < 0.001) significantly affected the survival of SPs. The vermiculite and perlite (1:1 (v/v)) treatment had 8.72 times (*p* < 0.001) the survival rate of perlite. Although BR had no significant effect on the survival of SPs (*p* = 0.062), the survival rate of BR 1 µg/L was 3.14 times (*p* < 0.05) that without BR. Of all the spectral treatments, the 8R2B treatment showed the greatest difference from cool white fluorescent, with a survival rate 5.71 times (*p* < 0.01) higher than that of white light (Table S4).

This study revealed that the optimal enhancement for *P. thunbergii* SPs was 20 g/L sucrose, 1/2 WPM liquid medium, and a culture substrate of vermiculite and perlite (1:1 (v/v)), which assisted the spectrum treatment. As for the unrooted SPs, a rooting medium of 1 1 µg/L BR was favorable for their growth. Following this acclimatization protocol, the survival rate of the somatic *P. thunbergii* plants transferred from the forcing house to the field was up to 90.94% (data not shown).

## Discussion

The results implied that the liquid medium (shoot, *p* < 0.001; root, *p* < 0.001), sucrose (shoot, *p* < 0.001; root, *p* = 0.002), and culture substrate (shoot, *p =* 0.03; root, *p* = 0.09) significantly impacted the growth of the SPs. For nematode-resistant *P. thunbergii* SPs, the optimal liquid medium was 1/2 WPM, followed by the GD medium. Additionally, the DCR medium was obviously not suitable for the growth of *P. thunbergii* SPs. Similarly, the slash pine (*Pinus elliottii* engelm) plantlets exhibited superior growth performance in the GD medium in contrast to the DCR medium [43]. Further analysis revealed that the concentrations of NO_3_^−^, NH_4_^+^, PO_4_^3−^, Mg^2+^, and Ca^2+^ in the DCR medium were not significantly different compared to the 1/2 WPM medium; however, the K^+^ content was only half that in 1/2 WPM medium (Table S2). It is well known that plant growth is modulated by K^[+ [44]]^. Tode and Luthen [45] reported that the enhancement of plant growth via both the fungal toxins fusicoccin and auxin required K^+^ uptake in maize coleoptiles. However, the K^+^ content of the DCR medium was the lowest of all media. Thus, we speculated that the poor growth performance of SPs under the DCR medium might have been related to the lower K^+^ content. Additionally, the 1/2 WPM medium contained sufficient minor elements (e.g., Mn, Zn, and Ni) in contrast to the other media. The Mn and Zn ions improved chlorophyll synthesis and photosynthesis, which resulted in the modulation of plant growth (e.g., tomato) [46, 47]. As a component of urease, Ni promoted the growth of *Caryophyllaceae* and *Cruciferae* plants by modulating the transport of nitrogen from the roots to the leaves [48, 49]. This suggested that the improved performance of SP in the 1/2 WPM medium may have been related to the concentration of trace elements.

In this experiment, the mixture of perlite and vermiculite at a 1:1 ratio as the culture substrate was superior to the perlite and vermiculite for only the enhancement of *P. thunbergii* SPs. The pH of the culture substrate affected the root differentiation, whereas its porosity was positively correlated with the plant height (e.g., *Cucumis sativus*) [50, 51]. The mixed culture substrate could mediate plant growth by regulating these elements. Similarly, the *P. elliottii* root length was improved with the mixture of perlite and vermiculite, compared to perlite only [49]. As for the carbohydrates, Bhattacharyya et al. [52] reported that 20 g/L sucrose efficiently enhanced the vertical growth and rooting of *Dendrobium nobile* plantlets. In this study, 20 g/L sucrose was also beneficial for the growth of *P. thunbergii* SPs; the needles of which were noticeably greener. However, the shoot and root lengths of *P. thunbergii* SPs were inhibited when the sucrose concentration reached 40 g/L. Zhu et al. [53] reported that photoassimilates were continuously increased when the *Hevea* leaf color changed from light to dark green, which was consistent with our research. Hdider [54] found the high concentrations of sucrose hindered photosynthesis in strawberry. Further, this was evidenced by the restricted growth of rice and maize when they accumulated higher concentrations of sugars in their leaves [55]. This implied that although sugar is essential for plant growth, it can be detrimental when excessive. Appropriate sucrose concentrations that promoted the growth of *P. thunbergii* SPs, might have been related to the regulation of photosynthesis.

Akin to the sucrose treatment, the 1 µg/L BR root treatment showed a similar enhancement for SPs. The BR mutants of *Arabidopsis* displayed a stronger dwarf phenotype and etiolated growth in the dark [56], while the application of BR increased the plant height of papaya [57]. Further, Gao et al. [58] reported the enhancement of photosynthesis in maize by foliar spraying with BR. The enhancement of *P. thunbergii* SPs via BR might have been related to the regulation of photosynthesis (the needles were darker green in 1 µg/L BR). However, the growth of *P. thunbergii* SP was inhibited when the BR concentration reached 10 µg/L; thus, the promotion of SPs using exogenous BR was beneficial at lower concentrations. The application of BR for roots at lower concentrations was more effective for plantlet growth (e.g., *Arabidopsis* species) [59, 60].

In this study, monochromatic red light promoted the elongation of taproots, while monochromatic blue light promoted shoot elongation. The optimal combination of the spectra for *P. thunbergii* SPs growth was red and blue at a 8R:2B ratio. Kvaalen [61] reported that red light wavelengths inhibited the elongation of Norway spruce (*Picea abies*) shoots compared to blue light and cool white fluorescent light. However, for lettuce, enhanced shoot growth resulted from exposure to red light rather than blue [62], which indicated that the light spectrum treatments on plant growth were variable between species. As is known, the photoreceptors for red light are the phytochromes (PhyA and PhyB), while the blue light photoreceptors are phototropins [63, 64]. In terms of light regulators, phytochrome interacting factors (PIFs) played a key role in the modulation of plant growth. In green seedlings, elongation is primarily mediated by PIF4 and PIF5 [65, 66]. In an *Arabidopsis thaliana* study, blue light stimulated the expression of phytochrome interacting factor4 (PIF4) and PIF5 in seedlings, whereas the PIF4 and PIF5 negatively modulated auxin signaling. For example, PIF4 and PIF5 repressed the expression of auxin-responsive marker genes IAA5 and GH3-LIKE [67]. This suggested that blue light promoted stem elongation in *Arabidopsis thaliana* by suppressing the expression of auxin genes. Although our study also revealed that blue light enhanced *P. thunbergii* SP elongation, the specific stimulating kinetics require further investigation. Our results also indicated that although the growth of the aboveground portions of the plantlets was suppressed, root development was promoted by red light, which has been reported elsewhere (e.g., Arabidopsis) [68–70]. Conifer seedlings are also known to respond to light spectrum treatments. Ranade et al. suggested that combined red and blue light treatments enhanced the biomass and fiber size of Scots pine, resulting in stable tree structures [30]. In general, light spectrum treatments have been recognized as an important factor for improving plant production and quality, which is extensively used in horticulture [71]. In this report, we discuss the effects of red, blue, and white light treatments during root and shoot development of nematode-resistant *P. thunbergii* plantlets following germination, which influenced root development, shoot elongation, and the survival rates of plantlets following transplantation. Our studies revealed that the light quality could be manipulated to obtain high quality plantlets (improved shoot and root growth).

All treatments to enhance the growth of *P. thunbergii* SPs contributed to its survival rate, which indicated that it was necessary to improve the growth and quality of somatic plants prior to transplantation. Luis et al. [72] considered that larger seedlings of *Pinus canariensis* may augment transplantation performance in contrast to smaller seedlings. Although no statistically significant correlation was identified between the rootstock ratio and survival rate in our study, the high survival rate was focused on a rootstock ratio of ~ 2 and 10. Interestingly, a survival rate of > 60% occurred in both the rootstock ratio of 10, with root lengths of between 10 and 30 cm, and the rootstock ratio of 2, with root lengths less of than 5 cm, which may have been related to their high quality. The correlation between root development and the survival rate indicated that root tips had the greatest impact on the survival rate, followed by root surface area, root volume, and root diameter, with the root length having the lowest. Thus, for the evaluation of plantlet quality, the root tips, root surface area, and volume were the main criteria. In short, this study demonstrated that enhancing the quality of SPs, particularly by stimulating their root development, is an effective strategy for improving their survival rate, which is a significant step forward for conifer afforestation strategies using SPs. Whereas, only one genotype was used in this study, we would replicate the study using more genotypes to ensure the reliable application of the promotion protocol.

## Conclusion

In this study, we initially articulated the acclimation protocols for nematode-resistant *P. thunbergii* SPs and monitored their survival rates following transplantation. The results indicated that a growth medium containing 20 g/L sucrose, 1/2 WPM liquid medium, and a culture substrate (vermiculite and perlite = 1:1) were optimal for improving the quality of *P. thunbergii* SPs. Moreover, blue light promoted the shoot length, whereas red light promoted the main root length. The survival rates of regenerated plants were enhanced by all treatments that improved the SP quality. This study investigated the factors involved in the transition from the heterotrophic (organic nutrient growth) to autotrophic (inorganic nutrient growth) stage of SPs. The culture conditions required to improve the quality and survival rates of SPs following transplantation were determined. This research provides a foundation for future research toward increasing the survival rates of plantlets and promotes the application of elite clones (nematode-resistant *P. thunbergii* plantlets) for afforestation.

## Electronic supplementary material

Below is the link to the electronic supplementary material.

Supplementary Material 1

## Data Availability

The data supporting the results are concluded in the article and supplementary information files.
