# Supplementary material for: Promoting the application of Pinus thunbergii Parl. to enhance the growth and survival rates of post-germination somatic plantlets
Source: BMC Plant Biol. 2023 Apr 12;23:195. doi: 10.1186/s12870-023-04175-1 (PMC10091573; doi:10.1186/s12870-023-04175-1)
Supplement: Supplementary file 1 — Supplementary Material 1 [file 12870_2023_4175_MOESM1_ESM.doc]

**Supplemental Information:**

| Table S1. Basic composition of liquid medium. | | | | | |
| --- | --- | --- | --- | --- | --- |
| Components | | Basic medium | | | |
|  |  | 1/2WPM  mg/L | GD  mg/L | LP  mg/L | DCR  mg/L |
| Major elements | NH4NO3 | 400 | 400 | 200 | 400 |
|  | KNO3 | 0 | 1515 | 909.9 | 340 |
|  | KH2PO4 | 170 | 90 | 136.1 | 170 |
|  | CaNO3 | 556 | 30 | 236.2 | 556 |
|  | Cacl2 | 96 | 150 | 0 | 85 |
|  | MgSO4 | 370 | 250 | 246.5 | 370 |
|  | K2SO4 | 1000 | 300 | 0 | 0 |
|  | MgNO3 | 0 | 0 | 256.5 | 0 |
|  | MgCL2 | 0 | 0 | 101.5 | 0 |
| Minor elements | KI | 0 | 0 | 4.1 | 0.83 |
|  | H3BO3 | 0 | 3 | 15.5 | 6.2 |
|  | MnSO4 | 44.6 | 10 | 10.5 | 22.3 |
|  | ZnSO4 | 17.2 | 3 | 14.7 | 8.6 |
|  | Na2MoO4 | 0.5 | 0.25 | 0.125 | 0.25 |
|  | CuSO4 | 0 | 0.25 | 0.173 | 0.25 |
|  | COCL2 | 0 | 0.25 | 0.125 | 0.3 |
|  | NICL2 | 12.4 | 0 | 0 | 0.3 |
| Iron salt | FeSO4 | 22.24 | 13.9 | 13.9 | 27.8 |
|  | EDTA | 29.84 | 18.65 | 18.7 | 37.3 |
| Organic additives | Nicotinic acid | 0.5 | 0.1 | 0.5 | 0.5 |
|  | VB6 | 0.5 | 0.1 | 0.5 | 0.5 |
|  | VB1 | 1 | 1 | 1 | 1 |
|  | Glycine | 2 | 0 | 2 | 2 |

| Table S2. Basic contents of main elements in liquid medium. | | | | |
| --- | --- | --- | --- | --- |
| Parameters | Basic medium | | | |
|  | 1/2WPM  mg/L | GD  mg/L | LP  mg/L | DCR  mg/L |
| NH_4_^+^ | 400 | 400 | 200 | 400 |
| NO_3_^-^ | 956 | 1945 | 1602.6 | 1296 |
| Total nitrogen | 1356 | 2345 | 1802.6 | 1696 |
| PO_4_^3-^ | 170 | 90 | 136.1 | 170 |
| K^+^ | 1170 | 1905 | 1050.1 | 510.83 |
| Ca^2+^ | 652 | 180 | 236.2 | 641 |
| Mg^2+^ | 370 | 250 | 604.5 | 370 |
| B^3+^ | 0 | 3 | 15.5 | 6.2 |
| Mn^2+^ | 44.6 | 10 | 10.5 | 22.3 |
| Zn^2+^ | 17.2 | 3 | 14.7 | 8.6 |
| Na^+^ | 0.5 | 0.25 | 0.125 | 0.25 |
| Cu^2+^ | 0 | 0.25 | 0.173 | 0.25 |
| NI^+^ | 12.4 | 0 | 0 | 0.3 |
| Fe^2+^ | 22.24 | 13.9 | 13.9 | 27.8 |

|  | | | | | | | | | |
| --- | --- | --- | --- | --- | --- | --- | --- | --- | --- |
| Table S3. The binary logistic regression analysis of survival rate for parameter in light treatment. | | | | | | | | | |
|  | | B | S.E | Wals | df | Sig. | Exp (B) | 95% C.I. for EXP(B) | |
|  |  |  |  |  |  |  |  | Lower | Upper |
| Step 1a | Treatments |  |  | 9.261 | 5.000 | 0.099 |  |  |  |
|  | Treatment (1) | 1.262 | 2.024 | 0.388 | 1.000 | 0.533 | 3.531 | 0.067 | 186.539 |
|  | Treatment (2) | 0.335 | 1.781 | 0.035 | 1.000 | 0.851 | 1.398 | 0.043 | 45.897 |
|  | Treatment (3) | 1.430 | 1.316 | 1.180 | 1.000 | 0.277 | 4.177 | 0.317 | 55.070 |
|  | Treatment (4) | -1.953 | 1.145 | 2.907 | 1.000 | 0.088 | 0.142 | 0.015 | 1.339 |
|  | Treatment (5) | -0.775 | 1.290 | 0.361 | 1.000 | 0.548 | 0.461 | 0.037 | 5.775 |
|  | Rootlength | 0.196 | 0.091 | 4.610 | 1.000 | 0.032 | 1.216 | 1.017 | 1.454 |
|  | SurfArea | -0.023 | 0.036 | 0.398 | 1.000 | 0.528 | 0.977 | 0.911 | 1.049 |
|  | AvgDiam | 1.181 | 0.307 | 14.851 | 1.000 | 0.000 | 3.258 | 1.787 | 5.942 |
|  | RootVolume | -0.359 | 0.200 | 3.229 | 1.000 | 0.072 | 0.698 | 0.472 | 1.033 |
|  | Tips | 0.458 | 0.084 | 29.803 | 1.000 | 0.000 | 1.581 | 1.341 | 1.864 |
|  | constant | -24.689 | 5.045 | 23.946 | 1.000 | 0.000 | 0.000 |  |  |

a. Variables entered on step 1: Treatment, Rootlength, SurfArea, AvgDiam, RootVolume, Tips. Treatment (categorical): code 0 = CK, 1 = B, 2 = 5R5B, 3 = 7R3B, 4= 8R2B, 5 = R. CK indicates cool white fluorescent; B indicates blue light; 5R5B indicates red light: blue light =5:5; 7R3B indicates red light: blue light =7:3; 8R2B indicates red light: blue light =8:2; R indicates red light. Survival rate (categorical): code 0 = dead, 1 = live.

| Table S4. The binary logistic regression analysis of survival rate for treatments. | | | | | | | | | |
| --- | --- | --- | --- | --- | --- | --- | --- | --- | --- |
| Treatments | | B | S.E | Wals | df | Sig. | Exp (B) | 95% C.I. for EXP(B) | |
|  |  |  |  |  |  |  |  | Lower | Upper |
| Step 1a | Medium |  |  | 15.7404 | 3.0000 | 0.0013 |  |  |  |
|  | Medium (1) | 0.9520 | 0.5314 | 3.2090 | 1.0000 | 0.0732 | 2.5909 | 0.9143 | 7.3420 |
|  | Medium (2) | 1.5950 | 0.5703 | 7.8229 | 1.0000 | 0.0052 | 4.9286 | 1.6117 | 15.0712 |
|  | Medium (3) | 1.7237 | 0.4534 | 14.4508 | 1.0000 | 0.0001 | 5.6053 | 2.3048 | 13.6321 |
|  | Substrate |  |  | 27.1101 | 2.0000 | 0.0000 |  |  |  |
|  | Substrate (1) | 0.4418 | 0.5455 | 0.6559 | 1.0000 | 0.4180 | 1.5556 | 0.5340 | 4.5316 |
|  | Substrate (2) | 2.1655 | 0.4748 | 20.8012 | 1.0000 | 0.0000 | 8.7193 | 3.4381 | 22.1127 |
|  | BR |  |  | 5.5405 | 2.0000 | 0.0626 |  |  |  |
|  | BR (1) | 1.1451 | 0.4873 | 5.5233 | 1.0000 | 0.0188 | 3.1429 | 1.2094 | 8.1673 |
|  | BR (2) | 0.2671 | 0.4482 | 0.3550 | 1.0000 | 0.5513 | 1.3061 | 0.5426 | 3.1441 |
|  | LED treatments |  |  | 24.4667 | 5.0000 | 0.0002 |  |  |  |
|  | LED (1) | -0.4130 | 0.4588 | 0.8103 | 1.0000 | 0.3680 | 0.6617 | 0.2692 | 1.6262 |
|  | LED (2) | 1.3231 | 0.5033 | 6.9113 | 1.0000 | 0.0086 | 3.7551 | 1.4003 | 10.0698 |
|  | LED (3) | 0.9808 | 0.4751 | 4.2625 | 1.0000 | 0.0390 | 2.6667 | 1.0510 | 6.7663 |
|  | LED (4) | 1.7430 | 0.5540 | 9.8967 | 1.0000 | 0.0017 | 5.7143 | 1.9291 | 16.9264 |
|  | LED (5) | 1.3231 | 0.5033 | 6.9113 | 1.0000 | 0.0086 | 3.7551 | 1.4003 | 10.0698 |
|  | Genotyoe (1) | -1.4518 | 0.3656 | 15.7663 | 1.0000 | 0.0001 | 0.2342 | 0.1144 | 0.4794 |
|  | Sucrose |  |  | 26.8179 | 3.0000 | 0.0000 |  |  |  |
|  | Sucrose (1) | 1.8648 | 0.4585 | 16.5389 | 1.0000 | 0.0000 | 6.4545 | 2.6276 | 15.8553 |
|  | Sucrose (2) | 1.5581 | 0.5604 | 7.7320 | 1.0000 | 0.0054 | 4.7500 | 1.5839 | 14.2451 |
|  | Sucrose (3) | -0.0000 | 0.5358 | 0.0000 | 1.0000 | 1.0000 | 1.0000 | 0.3499 | 2.8581 |
|  | Constant | -4.4358 | 0.8414 | 27.7903 | 1.0000 | 0.0000 | 0.0118 |  |  |

a. Variables entered on step 1: Medium, Substrate, Brassinosteroids (BR), LED treatments, Genotype, Sucrose. Medium (categorical): code 0 = DCR, 1 = LP, 2 = GD, 3 = 1/2 WPM; Substrate (categorical): code 0 = perlite, 1 = vermiculite, 2 = perlite, and vermiculite = 1:1; BR (categorical): code 0 = BR 0 µg/L, 1 = BR 1 µg/L, 2 = BR 10 µg/L; LED treatments (categorical): code 0 = CK, 1 = B, 2 = 5R5B, 3 = 7R3B, 4= 8R2B, 5 = R, CK indicates cool white fluorescent; B indicates blue light; 5R5B indicates red light: blue light =5:5; 7R3B indicates red light: blue light =7:3; 8R2B indicates red light: blue light =8:2; R indicates red light; Genotype (categorical): code 0 = 1539-1, 1 = 1637-2; Sucrose (categorical): code 0 = sucrose 10 g/L, 1 = sucrose 20 g/L, 2 = sucrose 30 g/L, 3 = sucrose 40 g/L; Survival rate (categorical): code 0 = dead, 1 = live.


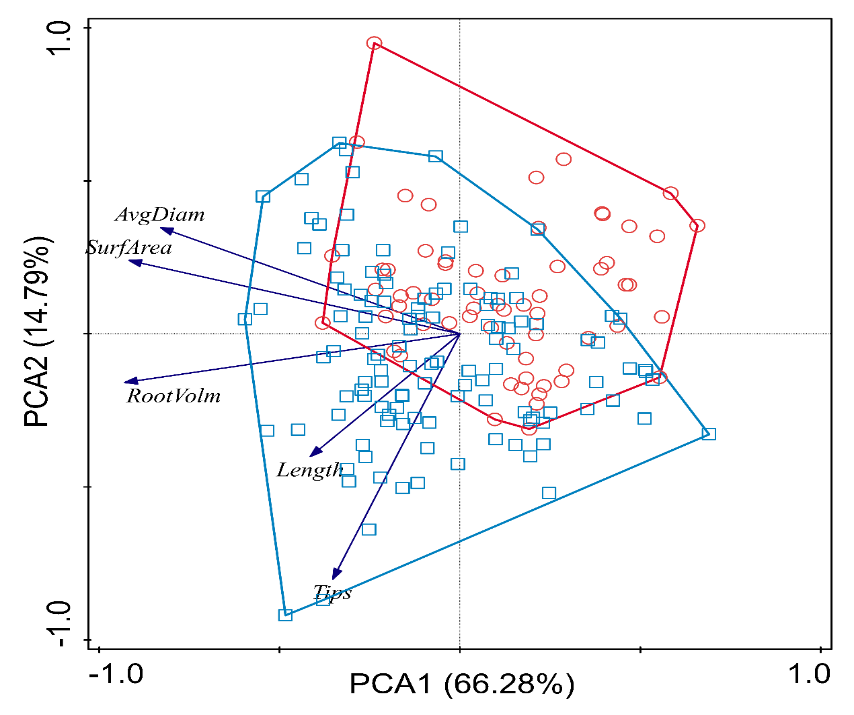


Fig. S1 Principal component analysis of plantlet growth for nematode-resistant *Pinus thunbergii.*

Red circles represent dead plantlets and blue rectangles indicate living plantlets.
